# Perioperative surgical outcome of conventional and robot-assisted total laparoscopic hysterectomy

**DOI:** 10.1186/s10397-017-1008-2

**Published:** 2017-04-25

**Authors:** W. J. van Weelden, B. B. M. Gordon, E. A. Roovers, A. A. Kraayenbrink, C. I. M. Aalders, F. Hartog, F. P. H. L. J. Dijkhuizen

**Affiliations:** 1 Department of Obstetrics and Gynecology, Rijnstate hospital, Wagnerlaan 55, 6815 AD Arnhem, The Netherlands; 2 Department of Obstetrics and Gynecology, Radboud University Nijmegen Medical Center, Geert Grooteplein-Zuid 22, 6525 GA Nijmegen, The Netherlands

**Keywords:** Total laparoscopic hysterectomy, Robot, Complication, Operating time

## Abstract

**Background:**

To evaluate surgical outcome in a consecutive series of patients with conventional and robot assisted total laparoscopic hysterectomy.

**Methods:**

A retrospective cohort study was performed among patients with benign and malignant indications for a laparoscopic hysterectomy. Main surgical outcomes were operation room time and skin to skin operating time, complications, conversions, rehospitalisation and reoperation, estimated blood loss and length of hospital stay.

**Results:**

A total of 294 patients were evaluated: 123 in the conventional total laparoscopic hysterectomy (TLH) group and 171 in the robot TLH group. After correction for differences in basic demographics with a multivariate linear regression analysis, the skin to skin operating time was a significant 18 minutes shorter in robot assisted TLH compared to conventional TLH (robot assisted TLH 92m, conventional TLH 110m, p0.001). The presence or absence of previous abdominal surgery had a significant influence on the skin to skin operating time as did the body mass index and the weight of the uterus.

Complications were not significantly different. The robot TLH group had significantly less blood loss and lower rehospitalisation and reoperation rates.

**Conclusions:**

This study compares conventional TLH with robot assisted TLH and shows shorter operating times, less blood loss and lower rehospitalisation and reoperation rates in the robot TLH group.

## Background

In recent years, it has become clear that laparoscopic hysterectomy offers a safe and feasible alternative to abdominal hysterectomy [1]. Patients with a laparoscopic hysterectomy have less complications, shorter hospital stays, and faster return to normal activities compared to abdominal hysterectomy [2]. When vaginal hysterectomy is not possible or not indicated, laparoscopic hysterectomy is the preferred alternative [2]. Despite the benefits of laparoscopic hysterectomy, abdominal hysterectomy remains the most common surgical approach, possibly because of the long learning curve and high-level laparoscopic skills necessary for a total laparoscopic hysterectomy [3–8]. Robotic surgery could overcome these difficulties, making laparoscopic surgery possible for more patients. The da Vinci system is the only registered robotic surgery system. It offers a three-dimensional vision, EndoWrist instruments, that mimics the human wrist and optimal ergonomics.

Up till now, the role of robotic surgery in total laparoscopic hysterectomy remains unclear. Studies comparing surgical outcome in conventional and robot-assisted total laparoscopic hysterectomy have shown mixed results [7, 9–11].

We performed the present study to evaluate the perioperative surgical outcome in a consecutive series of patients with conventional and robot-assisted total laparoscopic hysterectomy.

## Methods

### Design and setting

We conducted a retrospective cohort study comparing conventional total laparoscopic hysterectomy (TLH) with robot-assisted TLH. The study was performed between 2002 and 2014 in Rijnstate hospital Arnhem, a large teaching hospital in The Netherlands.

### Participants

All patients undergoing conventional or robot-assisted TLH with or without bilateral salpingo-oophorectomy were included in the study. We included all patients from the introduction of both techniques in our hospital onwards.

Patients with benign as well as malignant indications were eligible. Benign indications included fibroids, dysfunctional blood loss, and adenomyosis. Malignant indications included only low-grade endometrioid endometrial carcinoma. Patients with other subtypes of endometrial cancer and cervical cancer or patients who needed surgical staging procedures were not included [12]. Whenever a vaginal hysterectomy was deemed possible, that operation was preferred to a conventional or robot-assisted TLH in benign indications. These patients were also not included in this study.

Follow-up was performed in the outpatient clinic 6 weeks after operation. Further follow-up visits were planned if complaints or complications arose. In case of a malignancy, follow-up was planned according to the national guideline [12].

The institutional research board approved the present study. All patients gave informed consent for being included in the study.

### Procedure

All patients had general anesthesia and received preoperative antibiotics (cefazolin and metronidazole). Conventional TLH was performed using one 10-mm port at the umbilicus or in the midline 1.5 cm above the umbilicus, one assistant 5-mm port, and one assistant 12-mm port. For robot-assisted TLH, one 10-mm port in the midline 1.5 cm above the umbilicus and two 8-mm and one 12-mm assistant ports were inserted. Closed and open introduction techniques were used according to the discretion of the gynecologist. The surgical technique for both procedures was similar and is published elsewhere [13]. For the conventional TLH, we used the Gyrus bipolar system (Gyrus ACMI, Southborough, MA, USA); for the robot-assisted TLH, we used the da Vinci system with bipolar and monopolar currency (Intuitive Surgical Inc., Sunnyvale CA, USA). For vaginal manipulation of the uterus, we used the Clermont-Ferrand uterine manipulator (Karl Storz GmbH & Co., Tuttlingen, Germany). The vaginal cuff was closed laparoscopically in the same way for both procedures using a Quill or V-Lock running suture or knotted Vicryl 0 sutures.

Four laparoscopic gynecologists performed all conventional and robot-assisted TLH procedures. All were experienced in other gynecological laparoscopic operations, most notably laparoscopic supracervical hysterectomies before starting with conventional total laparoscopic hysterectomies.

Gynecologists performing robot-assisted TLH were trained according to the guideline for implementation of a new surgical technique from the Dutch Society of Endoscopic Surgery [14] and completed robotic training provided by Intuitive Surgical.

### Outcome

Demographic data including age, body mass index (BMI), indication for surgery (benign or malignant), and previous abdominal surgery was recorded.

Surgical outcomes were as follows:

- Operation room (OR) time: from patient arrival to departure of the OR.
- Skin to skin time: total operating time from skin incision to closure of the skin wounds.
- Complications: scored as a major or minor complication [15] (Table 1).
- Rehospitalisations and reoperations in case of a complication, estimated blood loss (EBL), and length of hospital stay (LHS, hospital stay started on the day of operation).

**Table 1:** Description of complications: major and minor [15]

Major	Minor
Hemorrhage requiring transfusion	Hemorrhage not requiring transfusion
Hematoma requiring surgical drainage	Hematoma: spontaneous drainage
Bowel injury	Infection: chest, urinary, wound, pelvic
Bladder injury	Deep vein thrombosis
Ureteric injury	Others
Pulmonary embolus	
Conversion to laparotomy	
Wound dehiscence	
Fistula	

### Statistics

Comparisons of group characteristics were made using chi-square or Fisher’s exact test for differences in proportions and Mann-Whitney *U* test for non-parametric distributed variables.

To correct for baseline differences between the two groups, multivariate linear regression analysis was performed in which type of robot-assisted TLH, conventional TLH, BMI, uterus weight, previous abdominal surgery, and indication for surgery (benign or malignant) were entered as independent variables. Operation room time and skin to skin operating time were entered as dependent variables. To get meaningful regression parameters, BMI and uterus weight were centered at 20 kg/m^2^ and 80 g, respectively. A *p* value of 0.05 was considered statistically significant. Statistical analysis was performed using IBM SPSS Statistics (version 21).

## Results

### Patient characteristics

Two hundred ninety-four patients were included in the study: 123 in the conventional TLH group and 171 in the robot TLH group. BMI and positive history of abdominal surgery did not differ significantly between the two groups. Age, uterus weight, and indication did differ significantly. Patients in the conventional TLH group were significantly younger (conventional TLH median age 49 years, robot-assisted TLH median age 57 years, *p* 0.03), had significantly higher uterus weights (conventional TLH 145 g, robot-assisted TLH 114 g, *p* 0.04), and were operated for malignant indications less frequently (conventional TLH 45%, robot-assisted TLH 58%, *p* 0.04) (Table 2).

**Table 2:** Baseline characteristics of conventional and robot TLH patients

	Conventional TLH (*n* 123)	Robot TLH (*n* 171)	*p* value
Age, median, years (range)	49 (29–89)	57 (27–88)	0.03
BMI, median, kg/m^2^ (range)	27 (17–52)	28 (18–59)	0.12
Uterus weight, grams (range)	145 (20–470)	114 (37–1009)	0.04
Indication for surgery, *n* (%)			
Benign	67 (55%)	72 (42%)	0.04
Malignant	56 (45%)	99 (58%)	
History of abdominal surgery, *n* (%)			
Positive	42 (34%)	77 (45%)	0.07
Negative	80 (66%)	94 (55%)	

### Operation room time

The median OR time was 173 min in the robot TLH group and 190 min in the conventional TLH group (*p* = 0.14 not significant) (Table 3).

**Table 3:** Perioperative surgical outcome

	Conventional	Robot	*p* value
Operation room time (min), median (range)	190 (102–303)	173 (116–393)	0.14
Skin to skin time (min), median (range)	145 (43–250)	120 (72–302)	<0.001
Complications, *n* (%)			
Major	9 (7.3%)	7 (4.1%)	0.48
Minor	14 (11.4%)	19 (11.1%)	
No	100 (81.3%)	145 (84.8%)	
Conversion, *n* (%)	4 (3.3%)	3 (1.8%)	0.46
Rehospitalisation, *n* (%)	13 (10.6%)	7 (4.1%)	0.04
Reoperation, *n* (%)	7 (5.7%)	2 (1.2%)	0.04
Blood loss (ml), median (range)	100 (0–800)	25 (0–600)	<0.001
Blood transfusion, *n*	0	0	1.0
Length of hospital stay (days), median (range)	4 (2–41)	3 (2–8)	<0.001

In the multivariate linear regression analysis of the OR time, robot-assisted TLH was 9 min shorter compared to conventional TLH (141 min versus 150 m versus *p* 0.10, not significant). Furthermore, previous abdominal surgery, BMI, and uterus weight all had significant influences on the OR time. Patients with previous abdominal surgery had an increased OR time of 12.5 min (*p* 0.02). If the BMI was higher than 20 kg/m^2^, 2.1 min for every extra point of BMI was added (*p* < 0.001) to the OR time. If the uterus weight was higher than 80 g, 0.2 min for every gram of uterus weight was added to the OR time (*p* < 0.001). Benign or malignant indication for surgery had no significant influence on the OR time.

For example, a patient with a BMI of 20, a uterus weight of 80 g, and no previous abdominal surgery would have an OR time of 141 min if a robot-assisted TLH was performed or 150 min if a conventional TLH was performed.

The average patient in this study had a BMI of 27, a uterus weight of 128 g, and no previous abdominal surgery. The OR time in this patient would have been 165 min in the case of a robot-assisted TLH or 174 min in a conventional TLH. If that same patient would have a prior abdominal operation, an extra 12.5 min would have to be added to the OR times in both robot-assisted and conventional TLH.

### Skin to skin operating time

The median skin to skin time was significantly shorter in the robot group compared to the conventional group (120 versus 145 min *p* < 0.001) (Table 3).

A multivariate linear regression analysis of the skin to skin time showed a significant 18-min difference favoring the robot TLH group (robot-assisted TLH 92 m, conventional TLH 110 m, *p* 0.001). The skin to skin time increased 11.8 min for patients with previous abdominal surgery (*p* 0.02), 1.8 min for each point of BMI above 20 (*p* < 0.001), and 0.2 min for each extra gram of weight of the uterus over 80 g (*p* < 0.001).

Hence, a patient with a BMI of 27, a uterus weight of 128 g, and no previous abdominal surgery would have a skin to skin operating time of 114 min in the robot group or 132 min in the conventional group.

If that the same patient has a BMI of 35, the skin to skin operating time would have been 129 min in the robot group or 147 min in the conventional group.

### Complications, conversions, rehospitalisations, and reoperations

No significant differences could be found in complications or conversions (Tables 3 and 4). There were 16 major complications: seven in the robot group and nine in the conventional TLH group (Table 4). In the robot group, there were three conversions to laparotomy, one hemorrhage requiring transfusion, one bladder injury, one pulmonary embolus, and one would dehiscence. In the conventional group, there were four conversions to laparotomy, two postoperative fistulas, one bowel and one bladder injury, and one hemorrhage requiring transfusion. All conversions were performed for strategic reasons. The minor complications did not differ significantly as well (conventional group 14 (11.4%) versus robot group 19 (11.1%)) (Table 4).

**Table 4:** Encountered complications

	Conventional TLH	Robot TLH
Major complication	9	7
Hemorrhage requiring transfusion	1	1
Bowel injury	1	0
Bladder injury	1	1
Pulmonary embolus	0	1
Unintented conversion to laparotomy	4	3
Wound dehiscence	0	1
Vesicovaginal fistula	2	0
Minor complication	14	19
Hemorrhage not requiring transfusion	0	1
Infection: chest, urinary, wound, pelvic	12	12
Hematoma: spontaneous drainage	0	2
Deep vein thrombosis	0	0
Others	2	4

Rehospitalisations and reoperations were significantly more prevalent in the conventional TLH group. Rehospitalisation occurred in 13 cases (10.6%) in the conventional group compared to seven cases (4.1%) in the robot group (*p* 0.04). Reoperation was necessary in seven cases in the conventional TLH group (5.7%) and two cases in the robot TLH group (1.2%) (*p* 0.04).

### Estimated blood loss

The median EBL was 100 ml in the conventional TLH group compared to 25 ml in the robot TLH group (*p* < 0.001) (Table 3). After correction for differences in BMI, weight of the uterus, indication, and previous abdominal surgery, there was 70 ml more blood loss in the conventional TLH than in the robot TLH group (*p* < 0.001).

### Length of hospital stay

The median LHS was 4 days in the conventional TLH group and 3 days in the robot TLH group (*p* < 0.001). After correction for differences in BMI, weight of the uterus, indication, previous abdominal surgery, and age, the LHS was 0.74 day longer in the conventional TLH group compared to the robot TLH group (*p* < 0.001).

## Discussion

We conducted a retrospective cohort study to evaluate the perioperative surgical outcomes of conventional and robot-assisted total laparoscopic hysterectomy. This study shows that patients in the robot TLH group have faster skin to skin operating times, less blood loss, and lower rehospitalisation and reoperation rates. After correction for BMI, uterus weight, indication, and history of previous abdominal surgery, the advantages in operating time, estimated blood loss, and length of hospital stay persisted.

Although the skin to skin time was faster, the OR time did not differ significantly. This difference might be explained by more extensive preoperative positioning in the robot group. The positioning needs to be more precise in robot patients because of the impossibility to adjust the position of the patient after connection of the operating ports to the robot. In conventional TLH, small adjustments in positioning are possible during the operation. We hypothesized that the OR time in the robot TLH group would become shorter when less time was necessary for positioning in a more experienced operation team. Indeed, a comparison of conventional and robot TLH groups without the first 25 cases of robot-assisted TLH procedures showed a significant 15 min shorter OR time in the robot group (*p* 0.009).

Our analysis clearly shows how OR time and skin to skin operating time in conventional and robot-assisted TLH are influenced by BMI, weight of the uterus, and previous abdominal surgery. To our knowledge, no previous study has demonstrated this so clearly in robot-assisted TLH.

With this knowledge, one could hypothesize that advantages of robot-assisted TLH are more outspoken in a specific subset of patients. Unfortunately, this study was too small to allow any comparison of complications and operation times in more complicated cases like patients with a BMI above 40 kg/m^2^ or with multiple previous abdominal operations. Further research is necessary to identify specific populations that profit specifically from a robot-assisted TLH.

The operating times in our study correspond well with the literature [10, 11, 16]. Shorter operating times are reported as well, but they originate mostly from early adopters of robotic surgery or centers with more experience in laparoscopic hysterectomies [17–19].

When comparing operating times between conventional and robot-assisted TLH, most studies have found shorter operating times in conventional TLH [10, 16–18, 20]. A Cochrane review conducted by Liu et al. concluded that moderate quality evidence exists for longer operating times in robot-assisted TLH [21]. This finding is based on two randomized controlled trials with in total 148 patients, which is half the population of our study [17, 19]. Other studies have also found shorter operating times in robot-assisted TLH [11, 16].

The present study, among many others, was not powered to show differences in the complication rate. Major complications like bladder injury and vaginal cuff dehiscence occur in about 2 and 1% of conventional total laparoscopic hysterectomies [6, 15]. A prospective trial with enough patients to show significant differences in these complications has to our knowledge never been performed. Our results however do give some indications for safer surgery in the robot TLH group. First, the estimated blood loss is 70 ml lower in the robot TLH group compared to the conventional TLH group. One could argue that 70 ml hardly has any clinical significance, but on the other hand, it might mean that robot-assisted TLH confers lower operation risks [16]. Also, the lower rehospitalisation and reoperation rates in the robot TLH group suggest that a robot-assisted TLH might convey a lower risk of complications than a conventional TLH.

Strengths of this study include the high number of cases included, the correction for relevant confounders, and the fact that the two operations are compared in one hospital performed by the same group of gynecologists. Two important limitations need to be addressed. First, the demographics differed between the conventional and robot TLH groups. By using a multivariate linear regression analysis, we corrected for these differences. Second, there are limitations inherent to the retrospective design of this study. In our experience, the operation itself has not changed dramatically from 2002 to 2014. The length of hospital stay however has decreased for all patients in our hospital. The difference in length of hospital stay that we found in this study therefore has to be interpreted with care.

## Conclusions

In conclusion, this study compares conventional TLH with robot-assisted TLH and shows that patients with a robot-assisted TLH have shorter skin to skin operation times and blood loss. Complications are not significantly lower in robot-assisted TLH, but when a complication arises, chances of rehospitalisation and reoperation are lower in the robot TLH group.
